# Evaluating the psychometric properties of the Swedish version of the Impostor Profile scale (IPP30)

**DOI:** 10.3389/fpsyg.2024.1341406

**Published:** 2024-03-22

**Authors:** Vini Doshi, Marijn Antens, Daiva Daukantaitė

**Affiliations:** Department of Psychology, Lund University, Lund, Sweden

**Keywords:** impostor phenomenon, Swedish version of the Impostor Profile scale, IPP30, validation study, exploratory factor analysis, confirmatory factor analysis

## Abstract

The Impostor Profile scale (IPP30) is a recently developed tool designed to delve into the nuanced aspects of the Impostor Phenomenon (IP), a psychological phenomenon where individuals wrongly attribute their successes to external factors, discounting their own abilities and often feeling like frauds. This study aimed to assess the psychometric properties, including factor structure, internal consistency, and nomological validity, of the Swedish version of IPP30 (S-IPP30). In a sample of Swedish students (*N* = 1,010; 76.7% women; *M_age_* = 25.65, *SD_age_* = 6.43), Exploratory and Confirmatory Factor Analyses were conducted to scrutinize S-IPP30’s structure. The analyses supported a bifactor model with six specific factors and one overarching factor. However, two items in the scale displayed poor alignment with their intended subscales, adversely affecting the internal consistency of the two subscales. Consequently, a rephrasing of these items was suggested. The remaining four S-IPP30 subscales exhibited good internal consistency (Cronbach’s *α* = 0.76–0.90, McDonald’s *ω* = 0.77–0.91). Convergent validity was confirmed by largely replicating correlations among various S-IPP30 facets, the unidimensional IP measure, personality variables, and self-esteem, thereby accomplishing the goal of validating S-IPP30. This proposed modification of the two items requires further validation using a new sample to ensure its appropriateness and effectiveness in measuring the intended constructs.

## 1 Introduction

“No matter what we have done, there comes a point where you think, ‘How did I get here?’ When are they going to discover that I am, in fact, a fraud and take everything away from me?” shares Tom Hanks in an interview (Gross, 2016). In spite of winning two academy awards and having starred in over 70 films and TV shows, he shares that he too, like many others, often struggles with low confidence and a feeling of alienation.

The Impostor Phenomenon (IP), commonly observed in ambitious individuals, refers to the phenomenon where individuals wrongly attribute their accomplishments to external factors like luck, disregarding their own capabilities (Clance and Imes, 1978). This mindset persists independently from their actual success (e.g., Brauer and Proyer, 2022). Additionally, they consistently express a fear of being exposed as frauds (Clance and Imes, 1978; Bravata et al., 2020; Chrousos et al., 2020). Although the IP was initially described in relation to high-achieving women (Clance and Imes, 1978), a meta-analysis conducted by Bravata et al. (2020) revealed that the extent to which the IP is reported varies widely, regardless of achievements, gender, ethnicity, or racial background.

Since its inception, various scales have been developed and utilized to measure the IP. These scales, which are mentioned below, have been developed based on a specific conceptualization of the IP by its creators, thereby emphasizing particular aspects of the phenomenon. However, their main limitation lies in their unidimensional scoring method, as they fall short of capturing the unique aspects of the multidimensional characteristics associated with the IP (Brauer and Wolf, 2016; Mak et al., 2019; Ibrahim et al., 2021, 2022a; Walker and Saklofske, 2023). This limitation leads to diverse interpretations of the IP, as understanding the different aspects of the IP is primarily confined to total scores, rather than considering the entire profile with its multiple facets, as experienced by different individuals. To overcome this limitation, Ibrahim et al. (2021, 2022a) introduced a new multidimensional scale, known as the Impostor-Profile 30 (IPP30). This scale provides a more comprehensive understanding of how individuals experience various facets of the IP, allowing for a more nuanced assessment.

The aim of this study is to assess the psychometric properties of the Swedish version of the IPP30 (hereafter referred to as S-IPP30). Currently accessible only in German and English (Ibrahim et al., 2022a,b), translating it into other languages would serve a broader audience. Moreover, given the absence of a validated and publicly accessible scale in Swedish for measuring IP, validating the scale in this language holds significant scientific importance. This validation process includes confirming and contrasting the S-IPP30’s factor structure, as examined by Ibrahim et al. (2021, 2022a,b), and evaluating its other psychometric properties (such as internal consistency, and nomological validity) within a large sample of Swedish students.

### 1.1 Measuring IP

To effectively assess and analyze the IP and develop interventions to address it, a comprehensive measurement tool is essential. Over time, five scales have been developed and utilized to measure the IP: The Harvey Impostor Phenomenon Scale (Harvey, 1981), the Clance Impostor Phenomenon Scale (CIPS) (Clance, 1985), the Perceived Fraudulence Scale (Kolligian and Sternberg, 1991), the Impostorism Scale (Leary et al., 2000), and the recently introduced Imposter Phenomenon Assessment (Walker and Saklofske, 2023). High reliability and validity are observed across all these scales (Chrisman et al., 1995; French et al., 2008; Mak et al., 2019; Walker and Saklofske, 2023), with the exception of the Harvey Impostor Phenomenon Scale (Chrisman et al., 1995). Furthermore, the initial development of the four older scales was rooted in a unidimensional framework based on specific conceptualizations of IP by their creators. However, subsequent analyses by other researchers, such as Chrisman et al. (1995), Brauer and Wolf (2016), and Brauer and Proyer (2023) demonstrated that, for instance, a 3-factor solution provided the best model fit for the CIPS in both English and German versions. Nevertheless, the number of factors varied across different studies, with a 2-factor model identified as the most fitting by French et al. (2008) and Fujie (2010), while others, including Jöstl et al. (2012) and Simon and Choi (2018), argued for a unidimensional factor structure as the most appropriate. Despite the proposed multifaceted nature of the CIPS, it is frequently interpreted as unidimensional due to the prevalent use of total scores. This practice limits insights into the diverse facets of IP experienced by individuals (Mak et al., 2019). Consequently, varying interpretations of IP may emerge, as the understanding of its aspects is confined to total scores alone, neglecting the comprehensive profile encompassing multiple facets of the IP experienced by individuals.

In response to these limitations, Ibrahim et al. (2021, 2022a) developed the IPP30, a multidimensional self-report questionnaire from its inception. The IPP30 questionnaire was constructed based on theories and definitions of the IP proposed by Clance and Imes (1978), Harvey and Katz (1985), and Sakulku and Alexander (2011). Unlike the previously mentioned scales, the IPP30 provides not only a total score but also six subscale scores. The first subscale, *Competence doubt*, assesses self-doubt, fear of failure, and maladaptive perfectionism related to competence, particularly before engaging in a performance task. The second subscale, *Working style*, indicates tendencies toward procrastination (high scores) or precrastination (low scores), both of which are reflective of the Impostor Cycle described by Sakulku and Alexander (2011). The subscale *Alienation* evaluates feelings of inauthenticity and a propensity for impression management. *Other-self divergence*, the fourth subscale, examines the strain caused by meeting external expectations, with high scores indicating significant discrepancies between self-image and perceived external image. The fifth subscale, *Ambition*, measures the need for success and high self-expectations. Finally, the sixth subscale, *Need for sympathy*, measures agreeableness, conflict-avoidance tendencies, and reliance on others’ cooperation (Ibrahim et al., 2021, 2022a).

More recently, another multidimensional scale, the Imposter Phenomenon Assessment (IPA) (Walker and Saklofske, 2023), has been introduced. This scale offers a 54-item self-assessment with three subscale scores and a total score. While a statistical comparison between the two multidimensional scales has not been conducted yet, Walker and Saklofske (2023) noted some differences, with a key distinction being that the IPP30 may allow differentiation between individuals experiencing impostor feelings and those intentionally feigning their personality or achievements, as indicated by the *Alienation* subscale. Additionally, the IPP30 may be more economical, requiring fewer items than the IPA. These differences, coupled with other limitations, emphasize the critical need to carefully choose a measurement tool that aligns with the nuanced aspects and dimensions one aims to capture when assessing IP.

The IPP30 scale demonstrated a consistent six-factor structure in both its German (Ibrahim et al., 2021, 2022a) and English (Ibrahim et al., 2022b) versions. The internal consistency of the six IPP30 subscale scores was generally deemed acceptable to high, with *Competence doubt* exhibiting the highest internal consistency (*ω* = 0.92 and 0.93 in both the German and English versions, respectively). However, it is important to highlight that *Ambition* and the *Need for sympathy* demonstrated relatively low internal consistency in both versions of the scale, with values of 0.67/0.66 for *Ambition* and.67/0.57 for *Need for sympathy* in the German/English versions, respectively.

When examining the relationship between the subscale scores and gender, it was observed that the correlations were generally low. In the German sample (Ibrahim et al., 2022a), these correlations ranged from 0.14 for *Ambition* to 0.22 for *Competence doubt*, suggesting a slightly elevated level of IP among women. On the other hand, in the English sample (Ibrahim et al., 2022b), no significant correlation with gender was found (*r* = −0.10, *p* > 0.05).

The scale demonstrated overall good nomological validity. In the two German samples, all subscale scores, except for *Ambition* and *Need for sympathy*, consistently showed at least moderate positive correlations with the CIPS. The correlations ranged from 0.17 for *Ambition* to 0.82 for *Competence doubt*, as reported by Ibrahim et al. (2021). Moreover, anticipated associations were also observed with other indicators, including personality traits such as Neuroticism (with the strongest relationship found for *Competence doubt*, *r* = 0.68, *p* < 0.001), Conscientiousness (with the strongest relationship found for *Working style*, *r* = 0.71, *p* < 0.001), Extraversion (with the strongest relationship found for *Alienation*, *r* = −0.49, *p* < 0.001), and self-esteem (with the strongest relationship found for *Competence doubt*, *r* = −0.74, *p* < 0.001).

### 1.2 Present study

In the current study, we aim to investigate the psychometric properties of the Swedish version of the IPP30 in a student population. Specifically, we will examine its factor structure, internal consistency, and nomological validity. Expanding upon the research by Ibrahim et al. (2021, 2022a,b), we expect that the S-IPP30 will manifest a bifactor model with six specific factors and one overarching factor.

As part of the validation process, we also aim to establish the convergent and discriminant validity of the S-IPP30 by assessing its correlations with various measures. Firstly, we will explore the associations between the S-IPP30 subscales and the CIPS (Clance, 1985), a widely recognized instrument for assessing the IP. Consistent with earlier studies conducted by Ibrahim et al. (2021), our hypotheses posit that the S-IPP30 subscales will show positive correlations with the CIPS.

Furthermore, we will investigate the correlations between the S-IPP30 subscales and the Big Five personality traits, along with self-esteem, as these factors have been identified as relevant to the understanding of the IP based on prior research (Clance and Imes, 1978; Leary et al., 2000; Vergauwe et al., 2015; Bravata et al., 2020; Ibrahim et al., 2022a).

IP is strongly linked to depression, shame, and anxiety (Bravata et al., 2020), all stemming from the persistent fear of exposure as a fraud. Earlier studies also suggest a positive correlation between the IP and Neuroticism (Vergauwe et al., 2015; Ibrahim et al., 2022a), indicating a higher likelihood of experiencing the IP among individuals with elevated Neuroticism scores. Consequently, we anticipate positive correlations between Neuroticism and the S-IPP30 subscales.

Conscientiousness, characterized by qualities such as ambition, thoughtfulness, and competence, was found to exhibit negative correlations with the German version of the IPP30 subscales (Ibrahim et al., 2022a). This suggests that individuals experiencing high levels of impostor phenomenon seem to lack feelings of competence (Vergauwe et al., 2015). Thus, in line with the research, we anticipate negative correlations between Conscientiousness and the S-IPP30 subscales. Moreover, existing literature suggests a negative relationship between Extraversion and the IP. This is likely due to the IP being associated with negative affect (Leary et al., 2000), while individuals scoring high on Extraversion typically experience feelings of cheerfulness and optimism (Vergauwe et al., 2015). Building on prior research and findings by Ibrahim et al. (2022a), we anticipate finding negative relationships between Extraversion and the S-IPP30 subscales. Individuals high in Extraversion, characterized by sociability and positive affect, are less likely to experience the negative feelings associated with impostor phenomenon, such as doubts about their competence and feelings of alienation.

In line with findings in previous research demonstrating an inverse association between self-esteem and feelings of impostorism (Clance and Imes, 1978; Bravata et al., 2020), and considering the negative correlations found between the German version of the IPP30 subscales and self-esteem by Ibrahim et al. (2022a), we anticipate a negative relationship between self-esteem and the S-IPP30 subscales as well.

Lastly, in alignment with previous research conducted in both German and English samples (Ibrahim et al., 2022a,b), we expect a weak correlation between gender and the S-IPP30 subscales.

## 2 Materials and methods

### 2.1 Participants

A total of 1,022 students enrolled at Lund University at the Bachelor’s level and upwards responded to the survey sent to their student email addresses. Using the Mahalanobis Distance, 12 outliers with significant distances at the *p* < 0.001 level were identified and excluded. In the end, the final sample consisted of 1,010 participants (*M* = 25.65 years, *SD* = 6.43). Participants were randomly divided into two subsamples for the Exploratory and Confirmatory Factor Analyses, respectively. Subsample 1 consisted of 505 participants (*M* = 25.81 years, *SD* = 6.83) and Subsample 2 consisted of 505 participants as well (*M* = 25.48 years, *SD* = 6.04). Table 1 highlights the sample characteristics in detail.

**Table 1:** Sample characteristics.

Variable	Overall (*n* = 1,010)	Subsample 1 (*n* = 505)	Subsample 2 (*n* = 505)
Gender			
Female	775 (76.7%)	402 (79.6%)	373 (73.9%)
Male	219 (21.7%)	97 (19.2%)	122 (24.2%)
Other	16 (1.6%)	6 (1.2%)	10 (2.0%)
Nationality			
Swedish-born	943 (93.4%)	471 (93.3%)	472 (93.5%)
Other	67 (6.6%)	34 (6.7%)	33 (6.5%)
Education			
Bachelor’s studies	634(62.8%)	325 (64.4%)	309 (61.2%)
Master’s studies	285(28.2%)	141 (27.9%)	144 (28.5%)
Other	91 (9.0%)	39 (7.7%)	52 (10.3%)

### 2.2 Measures

#### 2.2.1 The impostor profile scale

The impostor profile scale (Ibrahim et al., 2021) consists of 30 items, organized into six subscales: *Competence doubt* (11 items; e.g., “*Despite past successes, I have a strong fear of failure*”), *Working style* (6 items; e.g., “*I tend to complicate things by procrastinating*”), *Alienation* (3 items; e.g., “*I rarely show my true self*”), *Other-self divergence* (4 items; e.g., “*People tend to overestimate my abilities*”), *Ambition* (3 items; e.g., “*Creating something significant is very important to me*”), and *Need for sympathy* (3 items; e.g., “*Being liked by others is important to me*”). Responses are recorded using a visual analog scale ranging from 0 (“does not apply in any aspect”) to 100 (“applies completely”).

In previous studies conducted by Ibrahim et al. (2021, 2022a,b), acceptable to good internal consistency was noted for five out of the six subscales, with *Cronbach’s α/McDonald’s ω* values ranging from 0.66/0.71 to 0.92/0.91. However, the Need for sympathy subscale exhibited a lower level of reliability (*α* = 0.67, *ω* = 0.50). The internal consistency of the S-IPP30 for this study is reported in the Results section.

#### 2.2.2 The Clance impostor phenomenon scale

The Clance impostor phenomenon scale (Clance and Otoole, 1988) is the first scale created to measure IP and is still most frequently used to measure the phenomenon (Mak et al., 2019). It contains 20 items and uses a 5-point Likert scale ranging from 1 (“not at all true”) to 5 (“very true”). A systematic review performed by Mak et al. (2019) found the CIPS to have a good internal consistency, with Cronbach alpha values ranging from *α* = 0.85–0.96. In the study, the CIPS showed an excellent internal consistency with *α* = 0.92.

#### 2.2.3 The Big Five inventory

The Big Five inventory (John and Srivastava, 1999) is a 44-item self-report personality inventory based on the Big five dimensions of personality measuring Agreeableness, Extraversion, Conscientiousness, Openness to Experience, and Neuroticism. The scale uses a 5-point Likert response ranging from 1 (“strongly disagree”) to 5 (“strongly agree”). The English BFI has previously reported good internal consistencies (*α* = 0.79–0.88) (John and Srivastava, 1999). The Swedish BFI also reflects good internal consistency (*α* = 0.73–0.84) (Zakrisson, 2010). In the study, the BFI showed good internal consistency (*α* = 0.71–0.81).

#### 2.2.4 The Rosenberg self-esteem scale

The Rosenberg self-esteem scale (Rosenberg, 1965) is a 10-item questionnaire comprising an equal number of positively and negatively worded statements. Respondents provide their answers on a 4-point Likert scale, ranging from 1 (“strongly agree”) to 4 (“strongly disagree”). The RSES demonstrates robust reliability with a Cronbach’s *α* coefficient of 0.91 (Eklund et al., 2018). In the study, the internal consistency for the RSES was also found to be *α* = 0.91.

### 2.3 Procedure

An email with an anonymous link to the survey through Sunet Survey, a survey tool that is procured by Lund University and intended for similar purposes, was sent to the students at Lund University, Sweden. The data traffic between the participant and the server is encrypted. The system complies with the GDPR and personal data assistant agreements exist. The students received detailed information about the study, emphasizing the confidentiality and anonymity of their responses. Prior to participating, all participants provided informed consent by checking a box on an online form, indicating their understanding of the study’s purpose and their agreement to participate.

The original IPP30 was developed in German by Ibrahim et al. (2022a). An English translation provided by the authors was translated and back-translated to Swedish. Two native Swedish and English speakers were involved in this translation process. Any discrepancies between the English translation provided by the authors and the back-translated version were discussed. Item 22 “I often behave little authentic” in the IPP30 (English), was unclear to the translators. The developers of the scale were contacted for clarification. The Swedish version of the IPP30 can be found in Supplementary Table S1.

### 2.4 Ethical statement

All participants in the study were given informed consent, ensuring that they were fully aware of the voluntary nature of their participation, their right to withdraw from the study at any time, and the assurance of data confidentiality. The process of obtaining informed consent adhered to the principles outlined in the Declaration of Helsinki. The study protocol received approval from the ethical committee at the Department of Psychology, Lund University. To maintain anonymity, participants were instructed to create a unique code that could be used to withdraw their data from the dataset after the study was completed. At the conclusion of the study, participants were provided with a debriefing form. This document contained detailed information about the study, its purpose, and the contact details of the researchers. The debriefing process ensured that participants were well-informed about the study and had the opportunity to seek additional information or clarify any concerns they may have had. By implementing these ethical practices, the study prioritized participant autonomy, confidentiality, and well-being in line with established guidelines and regulations.

### 2.5 Statistical analyses

Statistical analyses including model testing, descriptive analyses and correlations were performed using the R software (R Core Team, 2020) with packages “lavaan” (Rosseel, 2012), “psych” (Revelle, 2022), and “jmv” (Selker et al., 2022).

The data were first checked for outliers. Using the Mahalanobis Distance, 12 outliers with significant distances at the *p* < 0.001 level were identified and excluded. Descriptive statistics were then performed on the final data (*N* = 1,010) to evaluate the sample characteristics and perform other statistical analyses. We conducted an Exploratory Factor Analysis (EFA) using Principal-axis extraction with Promax-rotation and the Parallel Factor method in the R-package “jmv” (Selker et al., 2022) to find a factor solution for the S-IPP30 with item loadings >0.30 on specific factors. The following fit indices for a good/satisfying model fit were used: Comparative Fit Index (CFI) > 0.95/0.90, Tucker-Lewis Index (TLI) > 0.95/0.90, the Root Mean Square Error of Approximation (RMSEA) < 0.06/0.08, the Standardized Root Mean Square of Approximation (SRMR) < 0.08/0.10, the Akaike Information Criterion (AIC), and the Bayesian Information Criterion (BIC), where lower values indicate better fit (Hu and Bentler, 1999). In the second step, a Confirmatory Factor Analysis (CFA) using Robust Maximum-Likelihood Estimation in the R-package “lavaan” (Rosseel, 2012) to replicate the structure as found in step 1 (i.e., Exploratory Factor Analysis) and also by Ibrahim et al. (2021, 2022a). The same model fit indices as described for the EFA were used. To assess measurement invariance across genders, we compared more restrictive models to a less constrained model, focusing on changes in CFI (ΔCFI) and RMSEA (ΔRMSEA). Consistent with previous research (Chen, 2007; Sass, 2016), we considered ΔCFI ≤0.010 and ΔRMSEA ≤0.015 as indicators of the invariance assumption being met.

The internal consistency was estimated with the use of Cronbach’s α and McDonald’s ω levels, with a criterion value of *>*0.70 for acceptable consistency (DeVellis, 2003). Pearson correlations were used to evaluate the nomological validity by investigating the correlations between the S-IPP30 subscales and the other instruments described in the previous section.

## 3 Results

### 3.1 Exploratory factor analysis

Using the R-package “jmv” (Selker et al., 2022), exploratory factor analysis was performed on Subsample 1 (*n*_1_ = 505, excellent sampling adequacy, KMO = 0.89). Promax-rotation was used in combination with principal-axis factoring extraction and those items with a main factor loading smaller than 0.30 were hidden. Following the recommended use of parallel analysis (Braeken and van Assen, 2017; Holubova, 2023), a 7-factor model was derived with good fit (RMSEA = 0.04, 90% CI [0.03–0.04], TLI = 0.95, BIC = 1109.427, *χ*^2^_(246)_ = 421.81, *p* < 0.001). Each factor consists of 3–11 items; absolute factor correlations ranged from 0.02 to 0.53.

As a consequence of the low inter-factor correlation between Factor 7 and Factor 6 (0.02) and the fact that Factor 7 consisted of only one item (item 15, factor loading 0.63), an EFA with a fixed factor method was used to determine the fit of a 6-factor model. The 6-factor model fit was also good (RMSEA = 0.04, 90% CI [0.04–0.05], TLI = 0.94, BIC = −1161.499, *χ*^2^_(270)_ = 519.13, *p* < 0.001). The factor loadings for the 6-factor model can be found in Table 2, where Item 15 and Item 16 have factor loadings of less than 0.3. The results were similar to those found by Ibrahim et al. (2021). Item 16 (“*I am considered a very helpful person*”) loaded on the same factor Ibrahim et al. (2021) found, whereas Item 15 (“A job in which I had many subordinates would satisfy me.”) loaded on Factor 1.

**Table 2:** Exploratory factor loadings of the S-IPP30.

Item	Competence doubt	Working style	Alienation	Other-self divergence	Ambition	Need for sympathy
1	0.83					
10	0.71					
4	0.71					
23	0.68					
13	0.67					
5	0.65					
26	0.61					
30	0.58					
**14**	−0.54					
18	0.40					
21	0.30					
2		0.91				
17		0.89				
6		0.88				
**11**		−0.80				
24		0.74				
**29**		−0.51				
28			0.93			
25			0.86			
22			0.68			
20				0.87		
3				0.73		
27				0.69		
7				0.34		
8					0.77	
19					0.67	
15						
12						0.91
9						0.68
16						

*N* = 505. Bold, reverse item. Only factor loadings ≥ 0.30 are presented in the table.

### 3.2 Confirmatory factor analysis

A confirmatory factor analysis was conducted using Robust maximum-likelihood estimation. The R-package “lavaan” was used to perform calculations (Rosseel, 2012). Using the second subsample (*n*_2_ = 505) four models were evaluated and compared based on fit indices (see Table 3). The single-factor model demonstrated inadequate goodness of fit (CFI = 0.42; TLI = 0.38; RMSEA = 0.14; SRMR = 0.13). Conversely, the six-factor model (CFI = 0.91; TLI = 0.90; RMSEA = 0.06; SRMR = 0.06) exhibited acceptable fit across all indices, akin to the second-order factor model (CFI = 0.90; TLI = 0.90; RMSEA = 0.06; SRMR = 0.07). However, the bifactor model with six group factors and a general factor demonstrated superior fit (CFI = 0.93; TLI = 0.92; RMSEA = 0.05; SRMR = 0.05) and marginally outperformed both the six-factor model (ΔCFI = 0.02, ΔTLI = 0.02, ΔRMSEA = 0.01, ΔAIC = −155.7) and the second-order model in terms of goodness of fit (ΔCFI = 0.03, ΔTLI = 0.02, ΔRMSEA = 0.01, ΔAIC = −184.1).

**Table 3:** Goodness-of-fit indices for model comparison.

Model	*χ* ^2^	df	CFI	TLI	RMSEA (90%)	SRMR	AIC	BIC
1-factor model	4304.4	405	0.42	0.38	0.14 (0.13–0.14)	0.13	142,387.1	142,767.3
6-factor model	998.7	390	0.91	0.90	0.06 (0.05–0.06)	0.06	139,111.4	139,555.0
Second-order model	1045.1	399	0.90	0.90	0.06 (0.05–0.06)	0.07	139,139.8	139,545.4
Bifactor model	813.0	375	0.93	0.92	0.05(0.04–0.05)	0.05	138,955.7	139,462.8

*N* = 505. CFI, comparative fit index; RMSEA, root mean square error of approximation; SRMR, standardized root mean residual.

**Figure 1:**
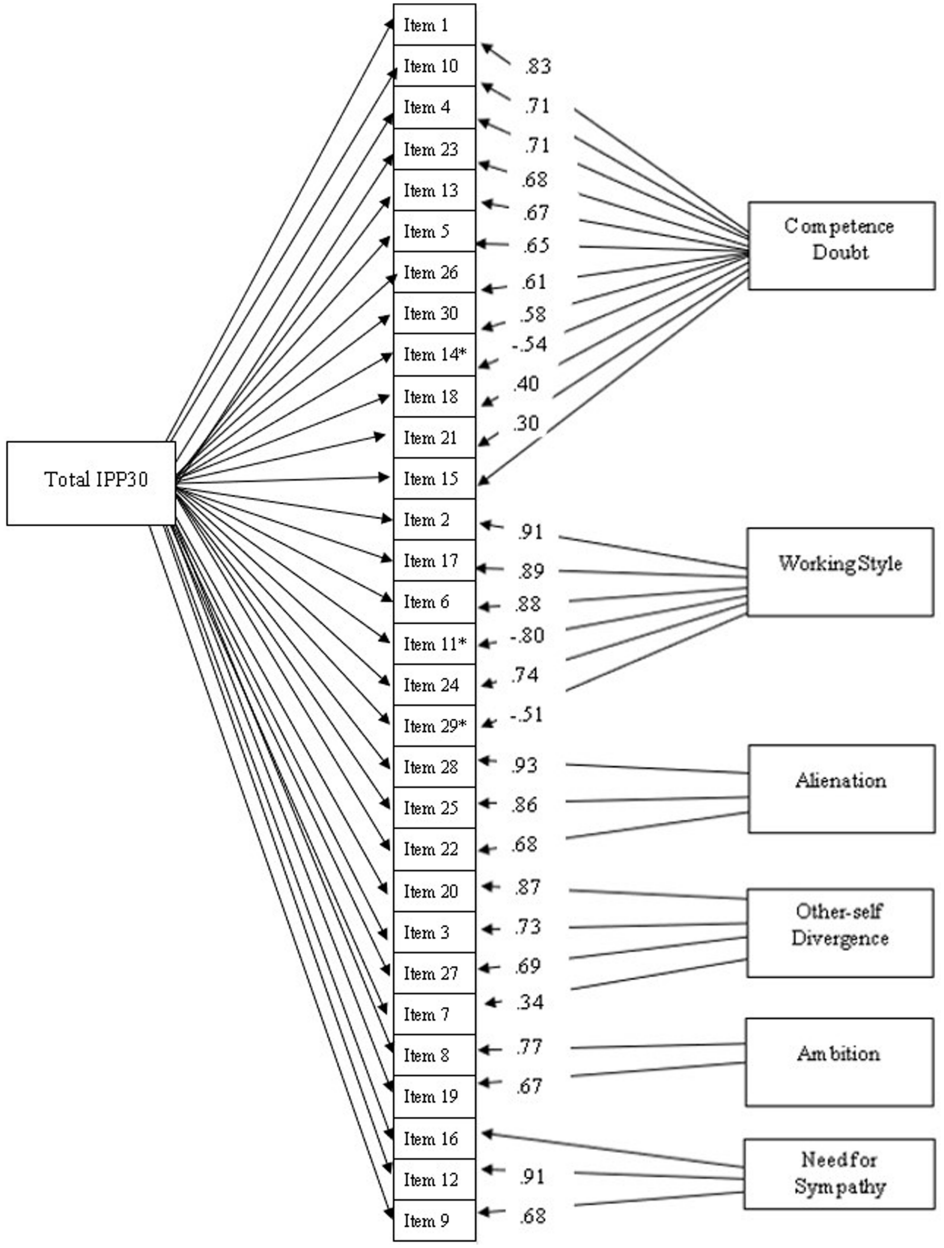
Factor loadings of the S-IPP30 items obtained from confirmatory factor analysis.

Notably, items 15 and 16 exhibited weak loadings even in the CFA, with standardized loadings of 0.11 and 0.23, respectively, though both were significant at the 0.001 level. All other items demonstrated loadings exceeding 0.40 on their respective factors and were statistically significant at *p* < 0.001 (Figure 1). Additionally, scalar invariance was confirmed for gender, as evidenced in Table 4, indicating directly comparable mean values across genders.

**Table 4:** Comparing configural, metric and scalar invariance across gender groups.

Invariance	*χ* ^2^	*df*	*p*	CFI	RMSEA	|Δ|	Δ*χ*^2^	*p* for Δ*χ*^2^	ΔCFI	ΔRMSEA
1. Configural	1251.01	670	<0.001	0.908	0.059					
2. Metric	1282.81	692	<0.001	0.907	0.059	|Δ| 2–1	31.79	0.081	0.001	0.000
3. Scalar	1313.44	714	<0.001	0.906	0.058	|Δ| 3–2	30.63	0.104	0.001	0.001

CFI, comparative fit index; RMSEA, root mean square error of approximation.

### 3.3 Internal consistency

The Cronbach’s alpha and McDonald’s omega coefficients for the six subscales demonstrated varying levels of internal consistency, ranging from *α* = 0.53 to 0.90 and *ω* = 0.64–0.91. As illustrated in Table 5, *Ambition* and *Need for sympathy*, each consisting of only three items and incorporating items 15 and 16—items that exhibited low loadings in both the earlier EFA and CFA—showed the lowest internal consistency values. Upon excluding these items, the internal consistency improved to satisfactory levels, yielding α/ω values of 0.72/0.73 and 0.74/0.74 for Ambition and Need for sympathy, respectively. A detailed overview of the internal consistency statistics is provided in Table 5.

**Table 5:** Reliability scores of the S-IPP30.

Scale/Item	*r* ^a^	*α* ^b^	90% CI	*M (SD)*	*ω* ^c^
**Competence doubt**		**0.89**	**0.87–0.90**	**60 (20)**	**0.89**
1	0.73	0.87		70 (27)	
4	0.72	0.87		68 (28)	
5	0.55	0.88		59 (32)	
10	0.73	0.87		54 (32)	
13	0.69	0.88		63 (30)	
14^*^	0.52	0.88		65 (28)	
18	0.52	0.88		53 (30)	
21	0.44	0.88		48 (33)	
23	0.62	0.88		67 (29)	
26	0.58	0.88		48 (34)	
30	0.61	0.88		68 (30)	
**Working style**		**0.90**	**0.89–0.91**	**54(27)**	**0.91**
2	0.84	0.87		55 (34)	
6	0.84	0.87		50 (34)	
11*	0.70	0.89		60 (33)	
17	0.81	0.88		53 (34)	
24	0.71	0.89		64 (31)	
29*	0.52	0.92		46 (28)	
**Alienation**		**0.87**	**0.86–0.90**	**33(27)**	**0.87**
22	0.69	0.86		31 (29)	
25	0.75	0.81		40 (32)	
28	0.80	0.76		29 (28)	
**Other-self divergence**		**0.76**	**0.73–0.80**	**40 (22)**	**0.77**
3	0.62	0.67		38 (26)	
7	0.38	0.80		38 (28)	
20	0.68	0.64		41 (29)	
27	0.58	0.69		44 (30)	
**Ambition**		**0.53**	**0.45–0.60**	**54 (20)**	**0.64**
8	0.43	0.21		74 (25)	
15	0.11	0.72		56 (30)	
19	0.45	0.13		33 (28)	
**Need for sympathy**		**0.59**	**0.52–0.65**	**76 (17)**	**0.68**
9	0.55	0.24		80 (23)	
12	0.49	0.34		76 (24)	
16	0.19	0.74		72 (20)	
**Total score**		**0.88**	**0.85–0.89**	**55 (14)**	**0.88**

*N* = 1,010. *Reverse scored items. ^a^Pearson’s r indicating the correlation between the item and the scale. ^b^Cronbach’s alpha; total values for subscales (bold); corrected subscale values when the item is dropped for each. ^c^Total omega. σ^2^ percentage of variance.

### 3.4 Nomological validity

The complete sample of 1,010 participants was utilized to assess the nomological validity of the S-IPP30 by examining the relationships between S-IPP30 subscale scores and the CIPS, the Big Five personality factors, as well as self-esteem.

Initially, we explored the intercorrelations among the S-IPP30 subscales. As displayed in Table 6, *Competence doubt* exhibited the strongest positive associations with the other S-IPP30 subscales, while the lowest intercorrelations were observed between *Ambition* and the remaining subscales.

**Table 6:** Inter-correlations between the S-IPP30 subscales.

Variable	2	3	4	5	6	7
Competence doubt	0.22^***^	0.39^***^	0.47^***^	0.11^***^	0.31^***^	0.86^***^
Working style	–	0.16^***^	0.17^***^	−0.08^*^	0.02	0.58^***^
Alienation		–	0.34^***^	0.01	0.05	0.54^***^
Other-self divergence			–	0.05	0.09^**^	0.62^***^
Ambition				–	0.19^***^	0.15^***^
Need for sympathy					–	0.35^***^
S-IPP30 total						–

*N* = 1,010. *p < 0.05 (two-tailed). **p < 0.01 (two-tailed). ***p < 0.001 (two-tailed).

Table 7 provides a concise overview of the correlations between S-IPP30 subscales and the other variables examined in the study. As expected, positive associations were found between CIPS and the S-IPP30 subscales, though their strength varied. Notably, the correlation with *Ambition* was the weakest (*r* = 0.08, *p* < 0.05), followed by the correlations with *Working style* (*r* = 0.22, *p* < 0.001) and *Need for sympathy* (*r* = 0.24, *p* < 0.001). In contrast, the most significant correlation was observed with *Competence doubt* (*r* = 0.82, *p* < 0.001).

**Table 7:** Correlations between S-IPP30 subscales and other studied variables.

Variable	Competence doubt	Working style	Alienation	Other-self divergence	Ambition	Need for sympathy	IPP30 total
CIPS total	0.82***	0.22***	0.39***	0.53***	0.08*	0.24***	0.77***
BFI subscales							
Neuroticism	0.67***	0.21***	0.34^***^	0.24^***^	< 0.01	0.19***	0.59***
Conscientiousness	−0.18***	−0.66***	−0.23***	−0.21***	0.14***	0.08**	−0.42***
Extraversion	−0.25***	−0.12***	−0.33***	−0.19***	0.20***	0.14***	−0.25***
Agreeableness	−0.08**	−0.11***	−0.27***	−0.15***	0.03	0.44***	−0.12***
Openness to experience	−0.07**	0.08*	−0.05	−0.12***	0.21***	0.03	−0.02
RSES total	−0.70***	−0.28***	−0.44***	−0.40***	−0.02	−0.11***	−0.68***
Gender	0.29***	−0.06*	−0.00	−0.02	0.12***	0.24***	0.15***

*N* = 1,010. CIPS, The Clance Impostor Phenomenon Scale; BFI, Big Five Inventory; RSES, Rosenberg Self-Esteem Scale. Gender: 1 = woman, 0 = man. *p < 0.05 (two-tailed). **p < 0.01 (two-tailed). ***p < 0.001 (two-tailed).

In the realm of personality traits, significant positive associations were observed between Neuroticism and all S-IPP30 subscales, except for *Ambition*. These associations exhibited varying strengths, ranging from *r* = 0.19, *p* < 0.001, for Neuroticism and *Need for sympathy* to a robust *r* = 0.67, *p* < 0.001, for Neuroticism and *Competence doubt.* Notably, Neuroticism displayed a very weak correlation (*r* < 0.01) with *Ambition*, suggesting a lack of substantial relationship between these traits in the study’s context.

Furthermore, Conscientiousness demonstrated strong and negative correlation with *Working style* (*r* = −0.66, *p* < 0.001), while Extraversion exhibited moderate and negative relationships with *Alienation* (*r* = −0.33, *p* < 0.001) and *Competence doubt* (*r* = −0.25, *p* < 0.001). Conversely, correlations with *Other-self divergence* were low (*r* = −0.19, *p* < 0.001), similar to those found with *Ambition* (*r* = −0.20, *p* < 0.001).

For the remaining two traits, Openness to experience and Agreeableness, no specific hypotheses were posited. Correlations with Openness were generally low, while *Need for sympathy* demonstrated a moderate correlation with Agreeableness (*r* = −0.19, *p* < 0.001).

## 4 Discussion

The results of the study conducted on the S-IPP30 scale largely replicated the bifactor model with six specific factors and one overarching factor, as reported in previous studies by Ibrahim et al. (2021, 2022a). However, it was observed that items 15 and 16 had low factor loadings in both exploratory and confirmatory factor analyses in the present study. These items also reduced the internal consistency of the *Ambition* and *Need for sympathy* subscales. One possible explanation for this finding is that the instrument in the present study was administered to a student population. Item 15 specifically inquired about feelings of job satisfaction with subordinates, which may not be relatable to most students who have limited experience in such positions. This discrepancy between our study and Ibrahim et al. (2021, 2022a) study, which included a working population, could be attributed to this reason. Furthermore, the other items measuring *Ambition* seem to assess different aspirations compared to Item 15. For example, “For me, it is very important to create something significant” and “Achieving something significant is what matters most to me in life” capture different aspects of Ambition. To address these issues and enhance the scale’s applicability to student populations, we propose a revision of item 15. A rephrased version could be: “I would be comfortable with leading a group of people to achieve a significant or important goal.” This revised statement aims to align more closely with the underlying construct of Ambition, ensuring its relevance to leadership positions focused on achieving goals and leading others.

The low factor loading on Item 16 may be attributed to its phrasing: “I am considered a helpful person.” In contrast to the other items in the same subscale measuring the Need for sympathy, which inquire about aspiring to possess specific values and behaviors, such as appearing sympathetic and being liked, Item 16 necessitates self-reflection. The Need for sympathy factor specifically refers to a need for popularity and an overreliance on the goodwill of others (Ibrahim et al., 2022a). As Item 16 does not measure the subscale in the same way as the other two items, we propose rephrasing it. For instance, a better measurement of the construct could be achieved with a revised item such as “It is important to be considered a helpful person.” It is imperative to validate this proposed revision of the two items using a new sample to ensure their appropriateness and effectiveness in measuring the intended constructs.

Another aspect that warrants further discussion regarding the IPP30 is the imbalance in the number of items across the subscales. The *Competence doubt* subscale consists of 11 items, while other subscales such as *Alienation, Ambition, and Need for sympathy* contain only 3 items. Given that the construct of *Competence doubt* aligns closely with the original IP definition (Bravata et al., 2020) and its underlying elements, as supported by the analyses, it is reasonable for this subscale to have the highest number of items. However, shortening this subscale would help prevent participant fatigue and achieve a more balanced measurement. Therefore, we suggest a critical evaluation of the items with the lowest factor loadings in this subscale and a reconsideration of which items should be included. Furthermore, the subscales with fewer items explained significantly less variance compared to those with more items. To address this issue, it might be beneficial to consider adding more items to these subscales in order to increase the variance explained by them.

Regarding the nomological validity of the S-IPP30, our findings align closely with both our hypotheses and prior research (Ibrahim et al., 2021, 2022a). Positive correlations were observed between the S-IPP30 subscales and the CIPS, a well-established measurement for the IP, indicating that the subscales adequately capture the IP construct. Furthermore, negative correlations were found between the subscales and Conscientiousness and Extraversion, while positive correlations were found with Neuroticism, providing further evidence for the good nomological validity of the scale. Interestingly, both the *Ambition* and *Need for sympathy* subscales showed positive correlations with Conscientiousness and Extraversion. The positive correlation between *Ambition* and Conscientiousness can be attributed to the fact that ambition is a core element of the Conscientiousness trait. Therefore, the positive association is not surprising. The positive correlation between Conscientiousness and *Need for sympathy* may be due to the shared association with thoughtfulness, as indicated in previous studies (Hoekstra et al., 2007). Additionally, high levels of Extraversion have been found to be related to higher popularity and a larger number of friends (Feiler and Kleinbaum, 2015), which explains the positive correlation between Extraversion and the *Need for sympathy* construct. The positive correlation between Extraversion and *Ambition* is likely due to the fact that Extraversion is associated with optimism (Sharpe et al., 2011), which could predict higher scores on the *Ambition* subscale.

Furthermore, consistent with our expectations, we discovered a negative association between self-esteem and the S-IPP30 subscales, aligning with previous research emphasizing low self-esteem as a fundamental aspect of the IP experience (Clance and Imes, 1978; Bravata et al., 2020; Ibrahim et al., 2021, 2022a). Lastly, the correlations between gender and the S-IPP30 subscales were notably low and closely resembled the findings reported by Ibrahim et al. (2022a), with women reporting higher levels of competence doubt and a greater need for sympathy, although these differences exhibited rather small effect sizes.

### 4.1 Strengths, limitations, and future directions

The sample size of this study contributes to the credibility and generalizability of the results within the student population. However, it is important to acknowledge that the findings may be limited to students exclusively, highlighting the need for future research to validate the S-IPP30 scale with diverse samples, including working professionals and individuals in different leadership roles. Furthermore, as mentioned earlier, a modification of two items displaying low loadings in both EFA and CFA, thereby compromising internal consistency, is warranted. Implementing this adjustment and testing the scale in more diverse samples would contribute to a more comprehensive understanding of its applicability across different populations.

Another limitation of this study is the lack of representation of individuals identifying with genders other than female or male. The existing research on the IP has predominantly focused on these two genders, and there is a dearth of information comparing the experience of the IP beyond this binary framework. Consequently, it is crucial for future studies to address this gap and explore the concept of the IP within the mentioned population. By doing so, we can gain a more comprehensive understanding of the IP and its impact across diverse gender identities.

### 4.2 Practical implications

The results of this study make significant contributions to the field of applied psychology by addressing the need for a measurement tool that caters to the culture, language, and population of Swedish-speaking individuals. Often, communities and populations are overlooked when it comes to measuring psychological constructs, and the IP is no exception. The lack of an available validated measurement tool for the Swedish population highlights the importance of this study’s contribution.

Furthermore, this study has implications for the existing perspectives on the IP in current literature. The findings emphasize the multidimensionality of the phenomenon, suggesting that future analyses should consider measuring the IP using a multidimensional approach rather than relying solely on a unidimensional total score. This insight adds nuance and complexity to our understanding of the IP and underscores the importance of capturing its various dimensions for a more comprehensive assessment.

## 5 Conclusion

The study findings demonstrated that the Swedish version of the IPP30 exhibited satisfactory psychometric properties. Both the EFA and CFA provided evidence of good model fit, indicating that the scale effectively measures the intended constructs, with the exception of two subscales that contained one item each, which loaded poorly on the expected factors. Rephrasing of these items was suggested, and further investigation in future research is warranted. The internal consistency of the scale, including both the total scale and subscales, was deemed satisfactory.

Additionally, a comprehensive examination of the correlations between the S-IPP30 and the CIPS, the Big Five personality traits, and self-esteem supported the scale’s good nomological validity. These correlations provided evidence for the expected relationships between the S-IPP30 subscales and external measures, strengthening the scale’s construct validity.

Future research should continue to explore and refine the scale, taking into account the identified limitations and potential improvements.

## Data availability statement

The raw data supporting the conclusions of this article will be made available by the authors, without undue reservation.

## Ethics statement

The studies involving humans were approved by the Etikkommittén at the Department of Psychology, Lund University. The studies were conducted in accordance with the local legislation and institutional requirements. The participants provided their written informed consent to participate in this study.

## Author contributions

VD: Conceptualization, Data curation, Methodology, Formal analysis, Investigation, Validation, Writing – original draft. MA: Conceptualization, Data curation, Formal analysis, Investigation, Methodology, Validation, Writing – original draft. DD: Conceptualization, Data curation, Methodology, Supervision, Writing – review & editing.
