# Supplementary material for: Evaluating the psychometric properties of the Swedish version of the Impostor Profile scale (IPP30)
Source: Front Psychol. 2024 Mar 22;15:1341406. doi: 10.3389/fpsyg.2024.1341406 (PMC10995337; doi:10.3389/fpsyg.2024.1341406)
Supplement: Supplementary file 1 [file Table_1.docx]

**Supplementary Material**

**Table S1**

*The S-IPP30 in Swedish*

| 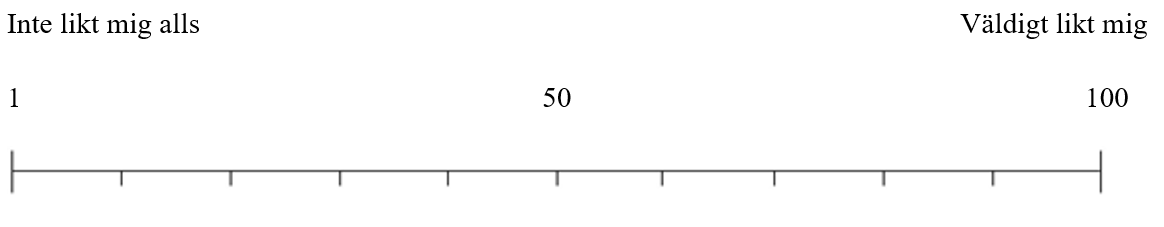 |
| --- |
| 1. Trots tidigare framgångar har jag en stark rädsla för att misslyckas. |
| 2. Jag gör många saker svårare för mig själv genom att skjuta på mitt arbete. |
| 3. Mina färdigheter är ofta överskattade. |
| 4. Jag tänker ofta att mina färdigheter inte är tillräckliga. |
| 5. Provsituationer är väldigt stressiga för mig. |
| 6. Jag skjuter ofta på att påbörja viktiga uppgifter. |
| 7. Mina kollegors förväntningar på mig är för höga. |
| 8. För mig är det väldigt viktigt att skapa något av betydelse. |
| 9. För mig är det viktigt att framstå som sympatisk. |
| 10. Min rädsla för att misslyckas förstör ofta det roliga med arbetet. |
| 11. Jag påbörjar mina uppgifter tidigare än vad andra gör. |
| 12. Det är viktigt för mig att bli omtyckt. |
| 13. Jag är inte övertygad om mina egna förmågor. |
| 14. Ett misslyckande är ingen anledning för mig att misstro mina förmågor. |
| 15. Ett jobb där jag har många underordnade skulle tillfredsställa mig. |
| 16. Jag uppfattas som en väldigt hjälpsam person. |
| 17. Jag blir oftast klar med viktiga uppgifter i sista minuten. |
| 18. För det mesta är jag missnöjd med kvaliteten på mitt arbete. |
| 19. Att åstadkomma något av betydelse är det viktigaste för mig i livet. |
| 20. Folk överskattar mig. |
| 21. När andra åstadkommer väldigt bra resultat, får det mig att känna mig otrygg. |
| 22. Jag beter mig ofta på ett sätt som inte är riktigt äkta |
| 23. Jag är rädd för att misslyckas, även om jag mestadels är framgångsrik. |
| 24. Jag distraherar ofta mig själv, även om jag har mycket att göra. |
| 25. Jag känner ofta att jag håller tillbaka min sanna personlighet. |
| 26. När jag är framgångsrik får jag ofta känslan att det kommer att följas av ett misslyckande. |
| 27. Jag anses ofta vara smartare än jag är. |
| 28. Jag är sällan mitt verkliga jag. |
| 29. Jag gör ofta de viktigaste uppgifterna först. |
| 30. Jag blir stressad när andra har höga förväntningar på mig |

*Note.* Items 15 and 16 exhibited low loadings in both the Exploratory Factor Analysis and Confirmatory Factor Analysis in the Swedish version of the IPP30. Modification is suggested before further use.
